# Research on Delamination Damage Quantification Detection of CFRP Bending Plate Based on Lamb Wave Mode Control

**DOI:** 10.3390/s24061790

**Published:** 2024-03-10

**Authors:** Quanpeng Yu, Shiyuan Zhou, Yuhan Cheng, Yao Deng

**Affiliations:** School of Mechanical Engineering, Beijing Institute of Technology, No. 5, Zhongguancun South Street, Haidian District, Beijing 100081, China; yuubit@163.com (Q.Y.); aslslcyh@163.com (Y.C.); 18701200802@163.com (Y.D.)

**Keywords:** Lamb wave mode control, phase time-delay method, CFRP bending plate, miniaturized linear comb transducer, signal difference coefficient, quantification of delamination damage

## Abstract

The carbon-fiber-reinforced polymer (CFRP) bending structure is widely used in aviation. The emergence and spread of delamination damage will decrease the safety of in-service bending structures. Lamb waves can effectively identify delamination damage as a high-damage-sensitivity detection tool. For this present study, the signal difference coefficient (*SDC*) was introduced to quantify delamination damage and evaluate the sensitivity of A0-mode and S0-mode Lamb waves to delamination damage. The simulation results show that compared with the S0-mode Lamb wave, the A0-mode Lamb wave exhibits higher delamination damage sensitivity. The delamination damage can be quantified based on the strong correlation between the *SDC* and the delamination damage size. The control effect of the linear array PZT phase time-delay method on the Lamb wave mode was investigated by simulation. The phase time-delay method realizes the generation of a single-mode Lamb wave, which can separately excite the A0-mode and S0-mode Lamb wave to identify delamination damage of different sizes. The A0-mode Lamb wave was excited by the developed one-dimensional miniaturized linear comb transducer (LCT), which was used to conduct the detection experiment on the CFRP bending plate with delamination damage sizes of Φ6.0 mm, Φ10.0 mm, and Φ15.0 mm. The experimental results verify the correctness of the simulation. According to the Hermite interpolation results of the finite-element simulation data, the relationship between the delamination damage size and the *SDC* was fitted by the Gaussian function and Rational function, which can accurately quantify the delamination damage. The absolute error of the delamination damage quantification with Gaussian and Rational fitting expression does not exceed 0.8 mm and 0.7 mm, and the percentage error is not more than 8% and 7%. The detection and signal processing methods employed in the present research are easy to operate and implement, and accurate delamination damage quantification results have been obtained.

## 1. Introduction

CFRP is increasingly used in aerospace, shipbuilding, and automotive industries due to its advantages of high specific strength and stiffness, light weight, and long fatigue life [1,2]. For example, in recent years, more than 50% of the structural weight of most aircraft has been made of CFRP, which reduces the aircraft’s total weight and fuel consumption [3]. CFRP is an orthotropic material made of carbon-fiber cloth layer by layer. Compared with isotropic materials, CFRP is prone to complex and inconspicuous damage forms during use [4], such as delamination, matrix cracking, and fiber rupture [5]. In addition, to meet the needs of various engineering applications, CFRP is usually made into different structures, such as tubes, beams [6], winglets [7], and bending structures [8]. Bending parts are commonly used aircraft structures [9], such as rectangular and C-shaped wing beams. The vulnerability of CFRP bending structures has been demonstrated [10]. Due to the structural characteristics of bent CFRP, the stress/strain concentration is easily induced in the bending area. As a result, the shape distortion and uneven distribution of carbon fibers occur in the bending area, ultimately leading to the appearance of damage. Delamination is the most common and critically damaging failure mode in CFRP bending structures [11,12]. Delamination usually occurs covertly within the CFRP structure. The structure’s stiffness, strength, and load-bearing capacity are significantly reduced due to the emergence and spread of delamination damage. When the size of delamination damage reaches a critical value, the bending CFRP structure will undergo severe overall failure [13]. Therefore, quantifying the delamination damage in the bending feature area is crucial for the safe and reliable operation of the bending CFRP structure during the service life.

As an active detection method, Lamb waves have the advantages of long propagation distance, small long-distance propagation attenuation, large coverage area, and high damage sensitivity [14]. Compared with other damage detection methods, Lamb waves have the characteristics of propagation along the plate and unique advantages for detecting bending plate damages. Lamb wave detection technology has emerged as one of the most promising approaches in non-destructive assessment applications, offering the capability to detect a range of damages (such as delamination, holes, etc.) within CFRP structures [15,16]. Lamb waves have two modes of symmetry and anti-symmetry due to their dispersion characteristics. Lamb wave components with different mode frequencies have unique sensitivities to delamination damage due to their propagation characteristics. When Lamb waves encounter delamination damage in the CFRP plate structure, phenomena such as scattering, transmission, and mode conversion will occur [17,18]. The Lamb waves propagate in each sub-layer above and below the layer, and sub-layers with different lay-up methods have different propagation velocities [19]. The propagation velocity, phase, and peak amplitude of Lamb waves will change due to the presence of delamination damage [20], and delamination damage with different sizes has different effects on Lamb wave propagation characteristics. Based on these properties of Lamb waves, delamination damage quantification detection can be achieved. Xianping Zeng et al. [21] detected the damage of large composite laminates using Lamb waves. The proposed damage contour algorithm using the convex envelope of damage reflection points and maximum inscribed n-polygons can accurately determine the quantification damage size. Jiahui Guo et al. [22] extracted the precise time-of-flight (ToF) of Lamb waves using the matching pursuit decomposition algorithm. The scattering source was identified on the elliptical trajectory obtained by the ToF of each sensing path. The Gaussian kernel probability density distribution of the scattering source provided an accurate characterization of the damage size. However, the damage quantification detection mentioned above was achieved through multiple sensors. It is meaningful to develop a simple and convenient damage quantification detection method.

Generally speaking, extracting information carried by Lamb waves based on time domain features is challenging. As a result, various signal analysis methods have been applied to Lamb waves to extract signal features. Pillarisetti et al. [23] obtained the power spectral density of A0-mode and S0-mode Lamb wave signals by performing fast Fourier transform and then quantifying the damage degree of CFRP based on the stress wave factor extracted from the power spectral density distribution. Research by Ying Tie [24] indicated that the amplitude of the relative acoustic non-linear parameters of Lamb waves increases with the rise in the delamination damage area. Guoqi Zhao et al. [25] employed PZT to excite and receive A0-mode Lamb waves to detect the delamination damage of the composite double cantilever beam. A0-mode Lamb wave signals were processed by Hilbert transform, Fourier transform, and continuous wavelet transform. Subsequently, the effective A0-mode Lamb wave non-linear parameters were extracted to characterize the delamination damage length. In addition, the time reversal imaging method was used to characterize the location and size of damage in CFRP plates, and satisfactory detection results were obtained [26]. However, utilizing the non-linear Lamb wave method for detection required experimental equipment with a higher sampling rate, and the execution of the time reversal method demanded more experimental steps and signal processing methods. Simple and feasible damage detection technology is of interest. As a simple and effective indicator, *SDC* can accurately measure the difference between Lamb wave signals. Sawant [27] and Raja B. [28] employed *SDC* as an indicator to precisely locate damage in CFRP.

In addition, the fundamental symmetric S0 and anti-symmetric A0 Lamb modes were used for practical detection [29]. Lamb waves in A0 mode and S0 mode have different propagation characteristics. At the same excitation frequency, the A0-mode Lamb wave has a lower propagation velocity and shorter wavelength than the S0-mode Lamb wave. Consequently, the A0-mode Lamb wave is more sensitive to damage [30,31,32]. However, it is difficult to analyze the signal of the A0-mode Lamb wave because it has higher dispersion and propagation attenuation than the S0-mode Lamb wave [33,34]. Ramadas et al. [35] investigated the interaction of A0-mode Lamb waves with delamination in the middle plane of CFRP laminates. The research shows that the A0-mode Lamb wave was converted into an S0-mode Lamb wave at the leading edge of the delamination. The S0-mode Lamb wave exclusively propagates within the sub-layer of the delaminated region and transitions back to the A0-mode Lamb wave upon exiting the delaminated area. This is the guiding significance for applying Lamb waves in nondestructive testing to study and analyze the sensitivity of fundamental mode Lamb waves to delamination damage.

To analyze the quantification detection ability of A0-mode and S0-mode Lamb waves on CFRP delamination damage, this paper introduces *SDC* to evaluate the sensitivity of A0-mode and S0-mode Lamb waves to delamination damage and quantify the delamination damage. The linear array PZT phase time-delay method was employed to realize the control and enhancement of single-mode Lamb waves. The quantification detection ability of single-mode Lamb waves toward delamination damage was investigated by simulation. The A0-mode Lamb wave was excited by the developed one-dimensional miniaturized LCT, which was employed to conduct the detection experiment on the CFRP bending plate with different delamination damage sizes. The relationship between the delamination damage size and *SDC* obtained by the finite-element simulation data can accurately quantify the delamination damage. The detection system framework of this research can detect damage in CFRP bending structures with angles in practical applications. Compared with other delamination damage quantification detection methods (such as the non-linear Lamb wave method and time reversal method), the method used in this paper is easy to operate and implement, and accurate delamination damage quantification results have been obtained.

## 2. Signal Processing Methods

The characteristics of the Lamb wave signal are altered due to the presence of damage. In this article, *SDC* was employed to evaluate the damage sensitivity of the Lamb wave. Concurrently, the damage size can be assessed by examining the *SDC* between the signal with damage information and the baseline signal without damage. *SDC* is defined as

(1) $$SDC=1-\frac{\sum_{i=1}^{m}\left(Sd_{i}-\mu_{Sd})(Sr_{i}-\mu_{Sr}\right)}{\sqrt{\sum_{i=1}^{m}{\left(Sd_{i}-\mu_{Sd}\right)}^{2}}\sqrt{\sum_{i=1}^{m}{\left(Sr_{i}-\mu_{Sr}\right)}^{2}}},$$

where *m* is the length of the signal sequence. *Sd_i_* and *Sr_i_* are the damage signal and the baseline signal, correspondingly, and *μ_Sd_* and *μ_Sr_* are the mean values of the corresponding signals, respectively. If there is no damage, the two signals are identical, resulting in an *SDC* value of 0. If the phases of the two signals are opposite, the *SDC* is close to 1.0.

To obtain the ToF of the Lamb wave, the continuous wavelet transform coefficients (*CWTCs*) of the Lamb wave signal are obtained through the application of wavelet transform. The wavelet transform is defined as follows:

(2) $$W_{f}(a,b)=\frac{1}{\sqrt{\left|a\right|}}\int_{-\infty }^{+\infty }S(t)\psi (\frac{t-b}{a})dt,$$

where *t* is time. *S*(*t*) is the signal sequence. $\psi (\frac{t-b}{a})$ is the wavelet basis function, where *a* is the scale factor, and its value is inversely proportional to the frequency. The small-scale factor can distinguish the high-frequency components of the signal sequence, while the large-scale factor can distinguish the low-frequency components of the signal sequence. Furthermore, *b* represents the displacement factor, denoting the time range during which a signal sequence of a specific frequency appears.

## 3. Finite-Element Simulation

Finite-element simulation technology can accurately calculate the propagation characteristics of Lamb waves in CFRP [36,37]. The three-dimensional finite-element analysis of Lamb wave propagation in CFRP was conducted in the Abaqus software (ABAQUS/CAE 2023) environment. In the finite-element simulation section, the selection of excitation signal frequency and mesh element size was undertaken. The simulation verified the control of the Lamb wave mode by the linear array PZT phase time-delay method. Finally, the simulation of delamination damage detection of the CFRP bending plate was carried out.

### 3.1. Finite-Element Simulation Model

**Figure 1:**
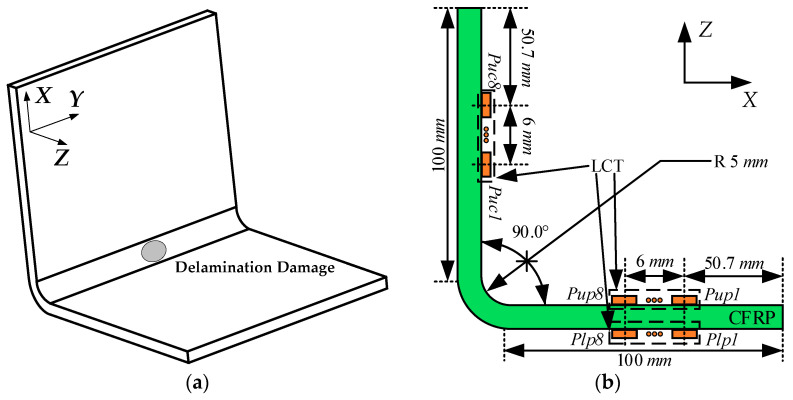
The structure of the CFRP bending plate and the location of delamination damage: (**a**) location of delamination damage; (**b**) structure size and location of LCT.

In this paper, the detection effect of A0-mode and S0-mode Lamb waves on delamination damage in CFRP bending plates was investigated by finite-element analysis. The finite-element calculation model and the location of delamination damage are shown in Figure 1a. The delamination damage was located at the center of the CFRP bending plate. Considering that the delamination shape might cause differences in the final damage detection capability, the present study set the delamination shape to be circular. The structural dimensions of the CFRP bending plate and the position of LCT on the CFRP bending plate are shown in Figure 1b. The bending radius *R* of the CFRP bending plate was 5.0 mm, and the thickness was 2.0 mm.

As shown in Figure 1b, the LCT used to excite the Lamb wave was pasted on the upper and lower sides of the CFRP plate. The relative position of delamination damage and LCT on the CFRP bending plate is crucial for the detection results. Therefore, the *Y*-axis coordinates of delamination damage and LCT were set to be consistent. The excitation elements of the LCT pasted to the upper side of the CFRP plate were numbered from *Pup1* to *Pup8*. The excitation elements of the LCT pasted to the underside of the CFRP plate were numbered from *Plp1* to *Plp8*. The LCT used to receive Lamb waves was pasted on the upper side of the CFRP plate, and the receiving elements of the LCT were numbered from *Puc1* to *Puc8*.

**Figure 2:**
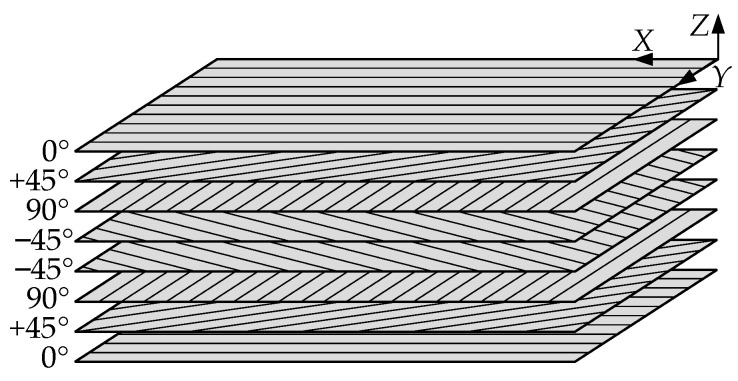
Ply direction of CFRP.

The shape, velocity, and amplitudes of Lamb waves were significantly affected by the ply orientation of CFRP [38]. The delamination damage quantification law obtained from CFRP with specific ply orientation did not apply to the delamination damage quantification in CFRP under other ply orientations. To verify the effectiveness of the proposed method for the delamination damage quantification of CFRP, the numerical simulation of [0°/45°/90°/−45°]_s_ quasi-isotropic CFRP was carried out [39]. The relationship between the ply direction and the coordinate axis is shown in Figure 2, and the CFRP material properties are listed in Table 1. In this paper, the mesh element type of CFRP was set to C3D8R.

**Figure 3:**
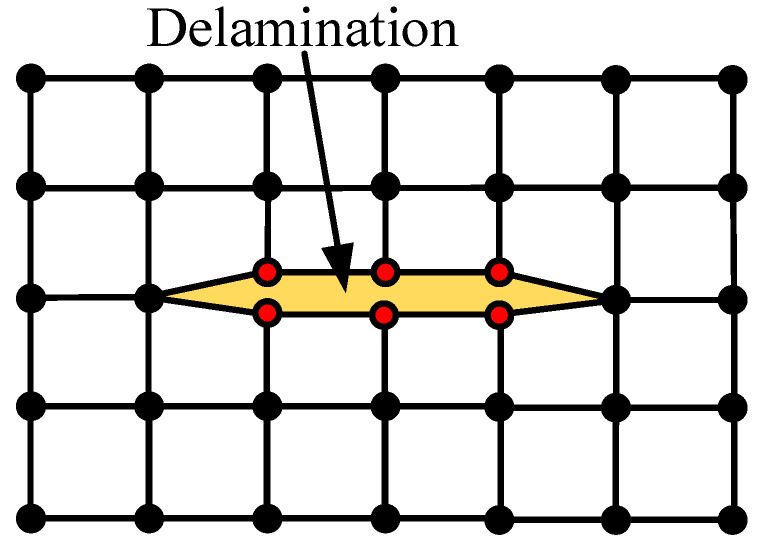
Schematic diagram of zero-volume delamination damage modeling method (within the delamination damage zone, two new red nodes are created from the original node, with the extent of the delamination damage exaggerated to emphasize the presence of these two new red nodes).

The sizes of delamination damage were Φ5.0 mm, Φ8.0 mm, Φ11.0 mm, Φ14.0 mm, Φ17.0 mm, and Φ20.0 mm. As shown in Figure 3, the detaching mesh node method was employed to generate delamination damage in the middle layer of the CFRP bending plate. A node was detached into two distinct nodes at the identical position to simulate zero-volume delamination damage. The CFRP bending plate without delamination damage was used as a reference. The time-domain signal of the CFRP bending plate without delamination damage was obtained by finite-element calculation as the reference signal.

PZT-5H was used as the excitation element and the receiving element, and its density was 7500.0 kg/m^3^. The stiffness coefficient, piezoelectric coefficient, and relative dielectric constant of PZT-5H are listed in Table 2, Table 3 and Table 4, respectively, and the absolute dielectric constant was 8.854 × 10^−12^ F/m. The mesh element type of PZT-5H was set to C3D8E in this article.

### 3.2. Selection of Excitation Signal Frequency

**Figure 4:**
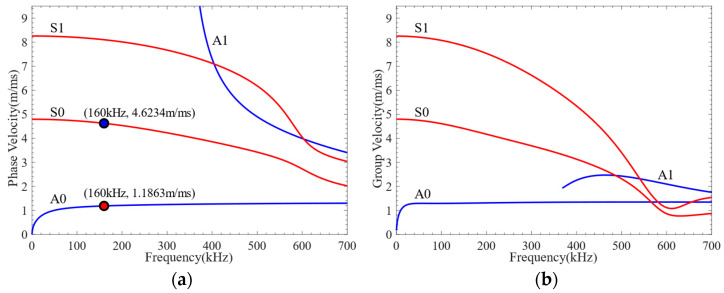
The dispersion curve of CFRP: (**a**) phase velocity; (**b**) group velocity.

**Figure 5:**
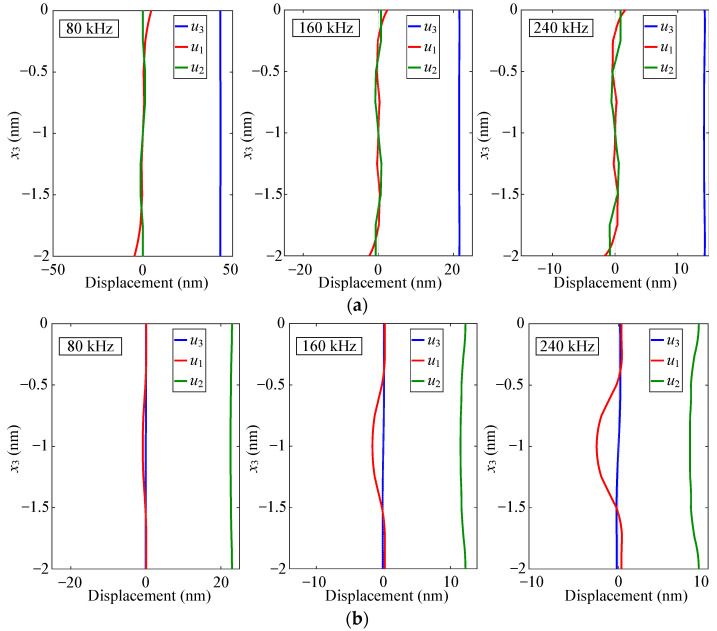
The through-thickness displacement profiles of Lamb waves propagating in CFRP: (**a**) A0-mode Lamb wave; (**b**) S0-mode Lamb wave.

**Figure 6:**
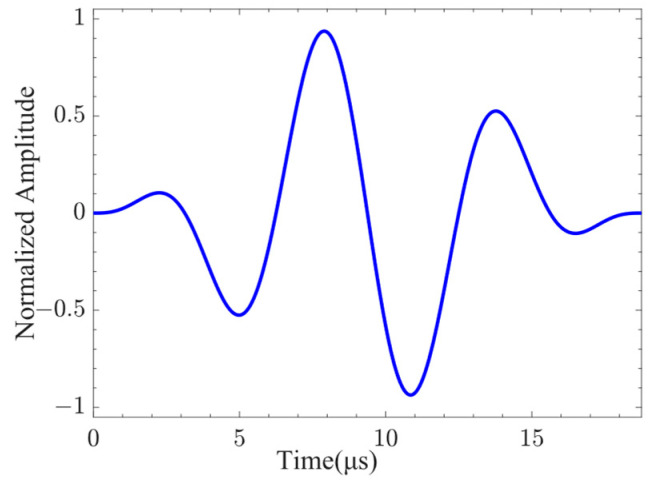
Excitation signal.

Lamb waves exhibit varying levels of sensitivity to damage at different frequencies. Before conducting damage detection, it is necessary to understand the dispersion characteristics of Lamb waves and select a more reasonable excitation frequency. However, there is no analytical solution to the dispersion equation for multilayer quasi-isotropic materials. The Dispersion Calculator [40] was used to calculate the phase velocity and group velocity dispersion curves of CFRP, as shown in Figure 4. The through-thickness displacement profiles of Lamb waves propagating in CFRP at frequencies of 80 kHz, 160 kHz, and 320 kHz are shown in Figure 5. It can be seen from the figure that the through-thickness displacement profile of CFRP is approximately the same below the frequency of 240 kHz. Consequently, a three-period narrowband Tone-Burst with a central frequency of 160 kHz modulated by the Hanning window was selected as the excitation signal [41]. The excitation signal is defined as follows:

(3) V(t)=0.5(1−cos(2πft/3))sin(2πft),

where the excitation frequency is *f* = 160 kHz. The waveform of the excitation signal is shown in Figure 6.

### 3.3. Selection of Mesh Element Size

The accuracies of the finite-element simulation results and computer time consumption are closely related to the mesh size. Theoretically, the smaller the mesh element size, the more accurate the calculation results will be, but the longer the computer time consumption [42,43]. To balance the contradiction between calculation accuracy and computer time consumption, it is meaningful to select the most suitable mesh element size for finite-element simulation. To ensure the simulation accuracy, the number of mesh nodes per wavelength must be guaranteed to exceed ten [41]. In this article, the group velocity of the A0 mode at the selected excitation frequency of 160 kHz was 1302.1 m/s. The minimum wavelength *λ*_min_ of the A0-mode Lamb wave signal propagating in the CFRP was calculated to be 8.1 mm. The mesh length *L* should satisfy the following equation:

(4) $$L\leq \lambda_{\min}/10=8.1/10=0.81\;\mathrm{mm},$$

To ensure the accuracy of the calculation results, this paper selected half of *L* as the maximum mesh element size for mesh independence verification. Four levels of mesh element size of 0.12 mm, 0.16 mm, 0.2 mm, and 0.4 mm were considered to verify the mesh element size independence of the finite-element simulation results. The ABAQUS/Explicit dynamic analysis solver was used to calculate the computational model with a total duration of 150 μs. The fixed time step for finite-element calculation was 10 ns.

**Figure 7:**
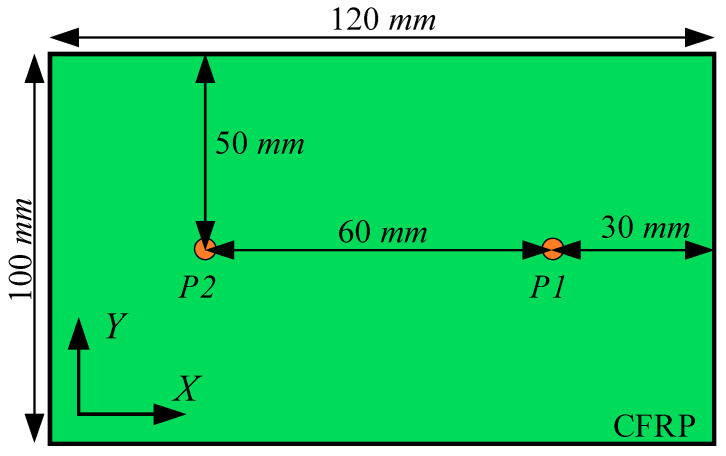
The computational model of mesh independence verification.

The CFRP plate calculation model is shown in Figure 7. Since the independence verification of the mesh element size only focuses on the dependence of the Lamb wave on the mesh element size during the propagation process, the Lamb wave excitation and reception method based on displacement loading was applied in the flat plate finite-element simulation. The excitation signal defined by Equation (3) was loaded at point P1, and the Lamb wave signals were received at point P2.

**Figure 8:**
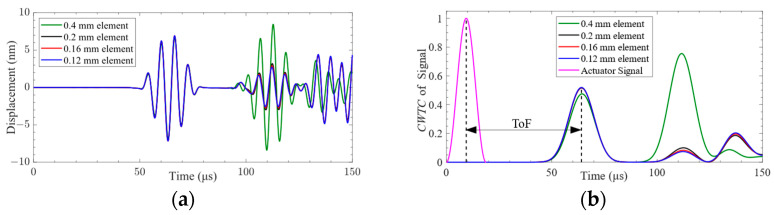
Lamb wave signal received at point P2: (**a**) time-domain signal; (**b**) *CWTC* of the signal.

**Figure 9:**
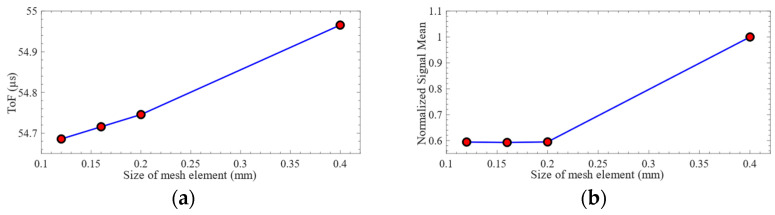
The characteristics of Lamb waves under different mesh element sizes: (**a**) ToF of the signal; (**b**) normalized signal mean.

The received time-domain signals and their *CWTCs* are shown in Figure 8. It can be seen from the figure that the time-domain waveform and *CWTC* of the mesh element size of 0.4 mm are very different from those of other mesh element sizes. The characteristics of the time-domain waveforms with mesh element sizes of 0.12 mm, 0.16 mm, and 0.2 mm are very similar. The characteristics of Lamb waves under different mesh element sizes are shown in Figure 9. It can be seen from the figure that the difference between the ToF obtained under each mesh element size is very small. Selecting the appropriate mesh element size was challenging based on the variation in ToF. The difference in the mean value of the signal obtained under the mesh element sizes of 0.4 mm and 0.2 mm is 68.2%. The difference in the mean value of the signal obtained under the mesh element sizes of 0.2 mm and 0.16 mm is 0.4%. Consequently, the mesh element with a size of 0.16 mm was selected in this paper to complete the subsequent research.

### 3.4. Mode Verification of Lamb Wave

**Figure 10:**
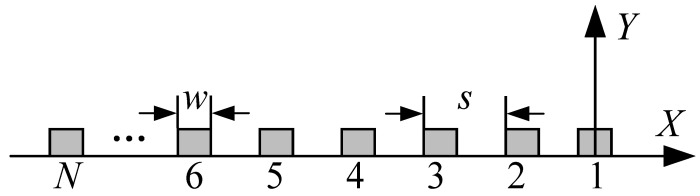
Schematic diagram of PZT array element distribution of LCT.

Lamb wave mode control can be achieved using an LCT. As shown in Figure 10, the LCT consists of a group of PZT array elements arranged in parallel at specific intervals. Control over Lamb wave modes was attained by adding phase time delay to the excitation signal of each PZT array element within the LCT. Assume that the LCT contains *N* PZT array elements. The distance between the PZT array elements is *s*, and the width of the PZT array elements is *w*. If the Lamb wave propagates along the negative direction of the *X*-axis, the phase time delay of the PZT array element numbered *N* is as follows:

*t_N_* = (*N* − 1)*t*_0_, (5)

where *t*_0_ = *s*/*c_p_* represents the phase time delay added between adjacent PZT array elements. And *c_p_* is the phase velocity of the Lamb wave, which can be obtained by calculating the phase velocity dispersion curve.

Each probe of the LCT developed in this article has eight PZT array elements. The distance between the PZT array elements *s* = 0.8 mm, and the width of the PZT array elements *w* = 0.4 mm. According to the phase velocity dispersion curve, the phase velocities of the A0 mode and S0 mode at 160 kHz can be obtained as 4623.4 m/s and 1186.3 m/s, correspondingly. The phase time delay *t_0_* added between adjacent PZT array elements of the LCT is 674.4 ns in the A0 mode and 173.0 ns in the S0 mode.

**Figure 11:**
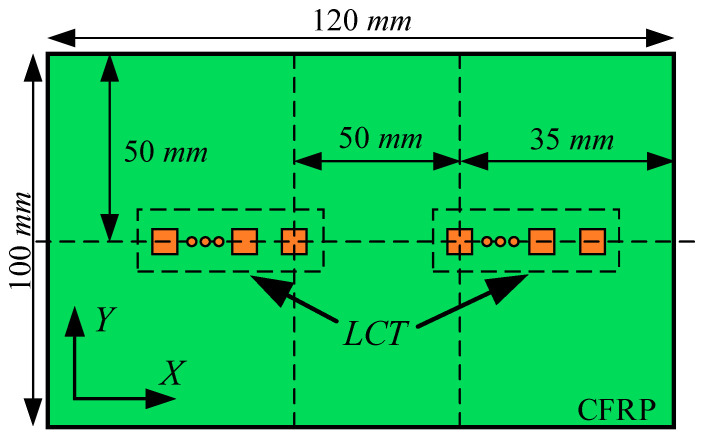
Computational model for Lamb wave mode control.

**Figure 12:**
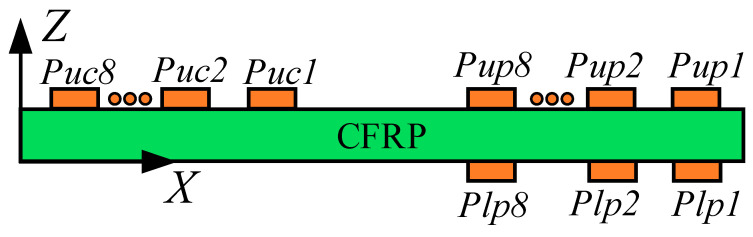
The paste schematic diagram of the LCT.

To verify the effectiveness of the Lamb wave mode control method in this article, the Abaqus implicit–explicit co-simulation technology was used to calculate the flat plate model, as shown in Figure 11. The paste position of the PZT array element of the LCT on the CFRP plate is shown in Figure 12. The total duration of finite-element calculation was 70 μs. The fixed time step of the finite-element calculation was 10 ns.

**Figure 13:**
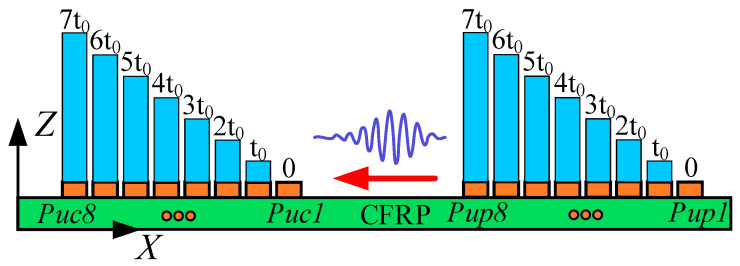
Phase time delay of excitation element and receiving element (the red arrow represents the propagation direction of Lamb wave).

The surfaces of the excitation element and the receiving element in contact with the CFRP were set as zero potential surfaces. The surfaces of the excitation elements parallel to the zero potential surfaces were set as the excitation surfaces for loading voltage. The surfaces of the receiving elements parallel to the zero potential surfaces were set as the receiving surfaces of the voltage. The phase time delay of each excitation element and the receiving element is shown in Figure 13. The phase time delay of the loading voltage remains consistent for the excitation element with identical *X*-axis coordinates, whether affixed to the upper or lower surfaces of the CFRP.

**Figure 14:**
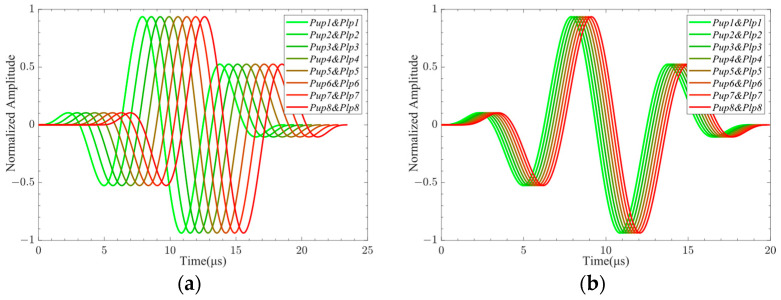
The excitation signals loaded on each excitation element: (**a**) The excitation signals were used to excite the A0-mode Lamb wave; (**b**) the excitation signals were used to excite the S0-mode Lamb wave.

The excitation signals loaded on each excitation element pasted on the upper and lower sides of the CFRP when the A0-mode Lamb wave was generated by controlling the phase time delay of the LCT are shown in Figure 14a. The voltage of the excitation surfaces of the excitation elements numbered *Pup1* to *Pup8* and numbered *Plp1* to *Plp8* was set to 500.0 V. The excitation signals loaded on each excitation element pasted on the upper and lower sides of the CFRP when the S0-mode Lamb wave was generated by controlling the phase time delay of the LCT are shown in Figure 14b. The voltage of the excitation surfaces of the excitation elements numbered *Pup1* to *Pup8* was set to 500 V, and the voltage of the excitation surfaces of the excitation elements numbered *Plp1* to *Plp8* was set to −500.0 V.

**Figure 15:**
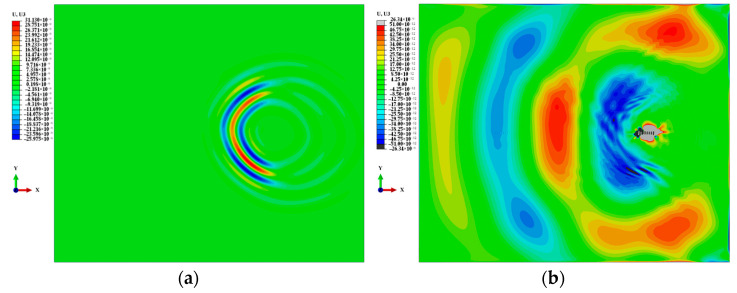
Propagation of Lamb wave signal: (**a**) A0-mode Lamb wave; (**b**) S0-mode Lamb wave.

The signal propagation of the A0-mode Lamb wave generated by LCT excitation at 27 μs is shown in Figure 15a. The signal propagation of the S0-mode Lamb wave generated by LCT excitation at 16.5 μs is shown in Figure 15b. It can be seen from the figure that the Lamb wave signals can be aggregated in the desired direction by controlling the LCT phase time delay. The change in phase time delay will produce different Lamb wave signal propagation phenomena. The wavelength of the S0-mode Lamb wave generated by LCT excitation is longer than that of the A0-mode Lamb wave.

**Figure 16:**
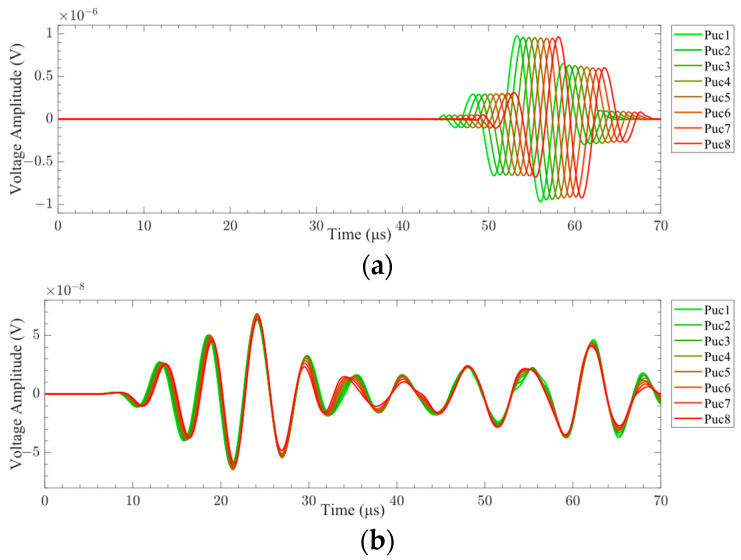
The surface average voltage signal received by the receiver: (**a**) A0 mode; (**b**) S0 mode.

**Figure 17:**
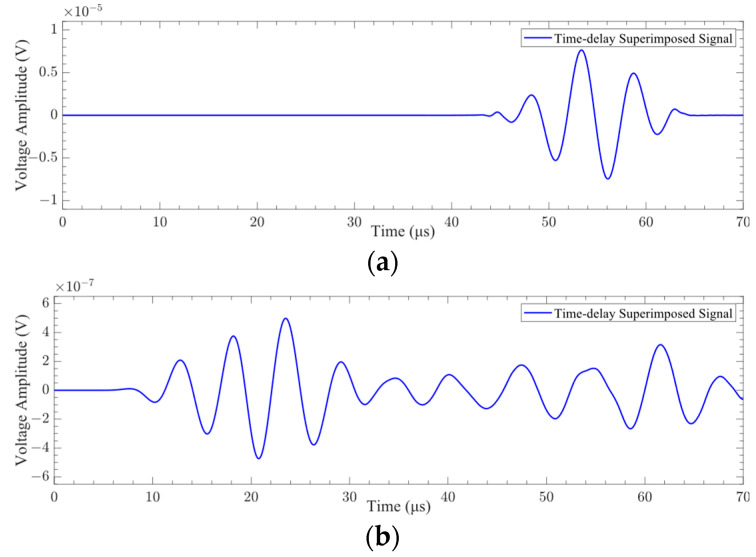
Time-delay superimposed signal: (**a**) A0 mode; (**b**) S0 mode.

**Figure 18:**
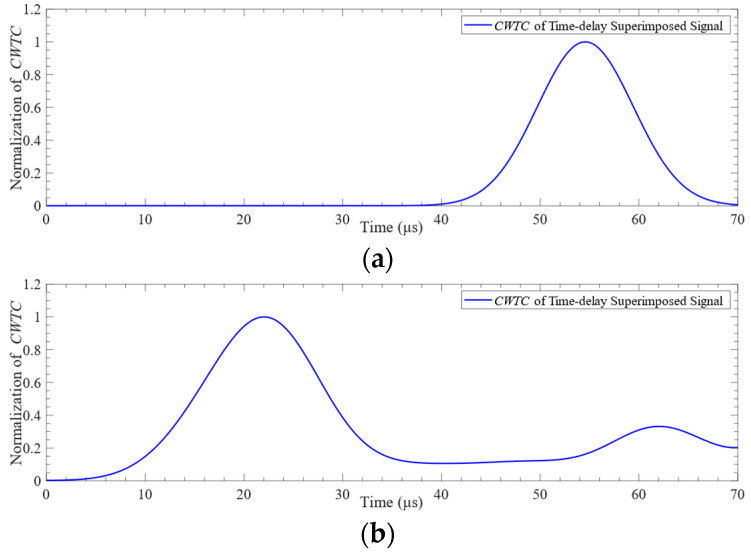
CWTC of time-delay superimposed voltage signal: (**a**) A0 mode; (**b**) S0 mode.

**Figure 19:**
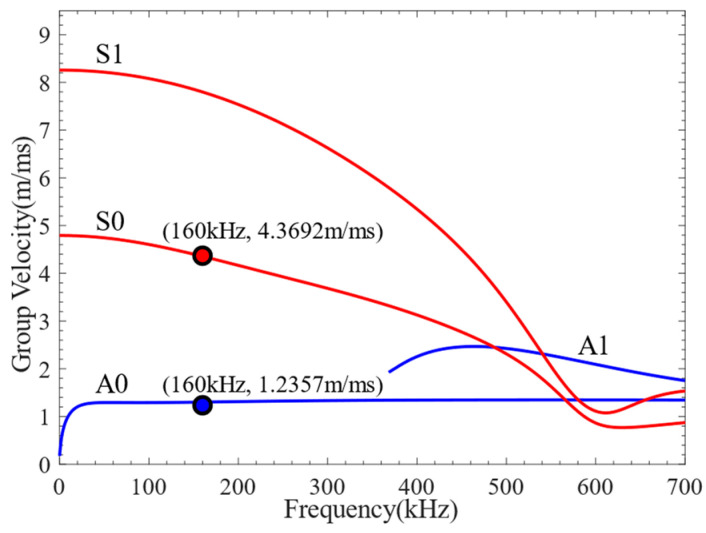
Verification of group velocity.

The A0-mode and S0-mode Lamb waves were generated by LCT excitation, and the surface average voltage signals received by each receiving element are shown in Figure 16. The time-delay superimposed processing of the surface average voltage signal received by the receiving element was carried out, and the time-delay superimposed voltage signals of A0-mode and S0-mode Lamb waves were obtained, as shown in Figure 17. It can be seen from Figure 16 and Figure 17 that different Lamb wave signals can be excited by changing the phase time delay of the excitation element. The time-domain distribution and amplitude of the Lamb wave signals were changed due to the different phase time delays. The continuous wavelet transform was applied to the time-delay superimposed voltage signals, and the *CWTCs* of the time-delay superimposed voltage signals were obtained as shown in Figure 18. It can be seen from the figure that the maximum point of the CWTC had changed, which was due to the change in time delay, resulting in the Lamb wave focusing in different modes. It can be seen from Figure 18b that the double wave was formed at 62 μs, which may have been due to the high wave velocity of the S0-mode Lamb wave, which was reflected at the boundary of the model. The ToF of Lamb wave propagation was obtained using the calculation method in Section 3.3, and the group velocity of the Lamb wave was calculated according to the propagation distance of the Lamb wave. As shown in Figure 19, the group velocities of A0-mode and S0-mode Lamb waves obtained by finite-element simulation were 1235.7 m/s and 4369.2 m/s at the frequency of 160 kHz, respectively. The group velocities of A0-mode and S0-mode Lamb waves calculated theoretically were 1302.1 m/s and 4359.4 m/s, respectively. As shown in Table 5, the absolute errors between the simulation results and theoretical calculation results of the group velocities of A0-mode and S0-mode Lamb waves were 66.4 m/s and 9.8 m/s, respectively, with percentage errors of 5.1% and 0.2%. The error between the simulation results and the theoretical calculation results was within the acceptable range. From Figure 16, Figure 17, Figure 18 and Figure 19, it can be summarized that the LCT can be employed to accurately excite A0-mode and S0-mode Lamb waves by changing the phase time delay of the excitation element.

### 3.5. Delamination Damage Detection of the CFRP Bending Plate

**Figure 20:**
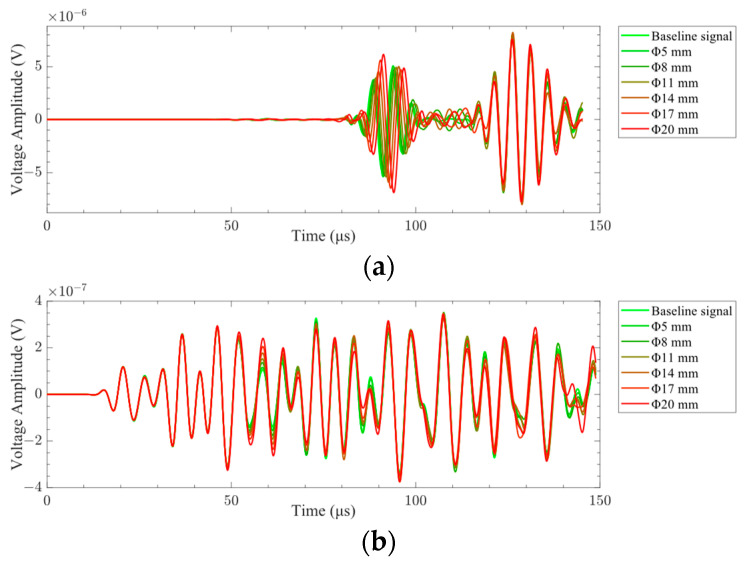
Time-delay superimposed voltage signal of delamination damage: (**a**) delamination damage time-delay superimposed voltage signal in A0 mode; (**b**) delamination damage time-delay superimposed voltage signal in S0 mode.

The finite-element calculation was carried out for the model shown in Figure 1. The time-delay superimposed voltage signals of different sizes of delamination damage obtained by finite-element calculation are shown in Figure 20. It can be seen from the figure that when A0-mode Lamb waves were used to detect delamination damage, the change in the size of layered damage had a significant impact on the time-delay superimposed voltage signal. The phase and amplitude of the time-delay superimposed voltage signal had changed due to the different sizes of the delamination damage. When the S0-mode Lamb waves were employed to detect the delamination damage, the influence of the delamination damage size on the time-delay superimposed voltage signal was non-significant.

**Figure 21:**
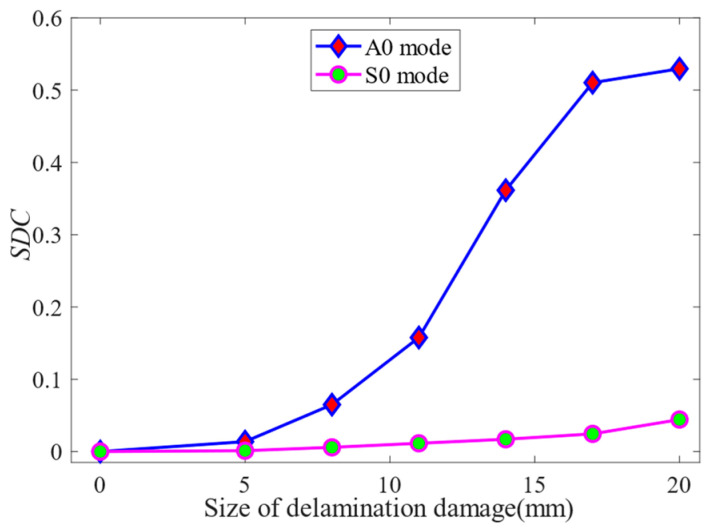
The *SDC* value of the time-delay superimposed voltage signal.

The *SDC* values of time-delay superimposed voltage signals with different sizes of delamination damage are shown in Figure 21. It can be seen from the figure that both in the A0 mode and in the S0 mode, the *SDC* value increases with the increase in the delamination damage size. Delamination damage can be quantified by analyzing the variation rule of *SDC* values with the size of the delamination damage. In addition, as shown in Figure 21, when the A0-mode Lamb wave is employed for delamination damage detection, the *SDC* value remains relatively constant in the case where the size of the damage is less than 5.0 mm or greater than 17.0 mm. This means that the *SDC* value does not significantly change with an increase in the size of the delamination damage within this range. In cases where the delamination damage size is less than 5.0 mm, the A0-mode Lamb wave is insensitive to the delamination damage because its wavelength is larger than the size of the damage. In cases where the delamination damage size exceeds 17.0 mm, the A0-mode Lamb wave cannot completely cover the large-scale delamination damage due to the small size of the PZT array element and its short distance from the delamination damage. When the size of delamination damage is between 5.0 and 17.0 mm, the *SDC* value increases significantly with the increase in delamination damage size, increasing from 0.013 to 0.51. The relationship between *SDC* value and delamination damage size is different in S0 mode and A0 mode.

In the case of delamination damage detection using S0-mode Lamb waves, when the delamination damage size is less than 20.0 mm, the *SDC* value gradually increases from 0.0 to 0.05 with the increase in the delamination damage size. This is because the S0-mode Lamb wave is poorly sensitive to delamination damage size due to its longer wavelength and faster wave velocity. According to the variation in *SDC* value with the size of delamination damage, it can be concluded that the A0-mode Lamb wave is more sensitive to delamination damage identification.

Simultaneously, considering that the amplitude of the time-delay superimposed voltage signal of the S0 mode was nearly 20 times lower than that of the A0 mode, it is plausible that the signal-to-noise ratio of the S0-mode Lamb wave signal excited in the experiment might be small. As a result, obtaining available S0 Lamb wave data during the experiment was challenging. Therefore, the A0-mode Lamb wave was excited for the experiment of delamination damage quantification detection.

## 4. Experiment

In the experiment section, a miniaturized LCT was developed based on the simulation model. The miniaturized LCT was used to excite and receive A0-mode Lamb waves. The Lamb wave phased array detection system was employed for verification experiments on the CFRP bending plate specimens with prefabricated delamination damage.

**Figure 22:**
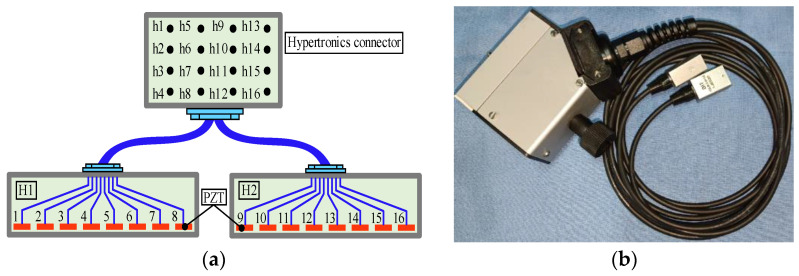
The structural diagram and product of miniaturized LCT: (**a**) the structure diagram of the miniaturized LCT; (**b**) the miniaturized LCT product.

A miniaturized LCT was developed to excite and receive Lamb waves of expected modes in experiments. The structure diagram of the miniaturized LCT is shown in Figure 22a. The width of the PZT array element and the distance between adjacent array elements were the same as the size set in the simulation. Hypertronics served as a connector for LCT, with pins numbered from h1 to h16. The two probes of the developed LCT shared a Hypertronics connector. The two probes of the miniaturized LCT were numbered H1 and H2, and each probe contained 8 PZT elements. The PZT array elements of probe H1 were numbered 1 to 8, and those of probe H2 were numbered 9 to 16. Probe H1 was used to excite Lamb waves, while probe H2 was used to receive them. The PZT array elements numbered 1 to 8 were connected to the pins numbered h1 to h8, respectively. Similarly, the PZT array elements numbered 9 to 16 were connected to the pins numbered h9 to h16, respectively. Each array element could individually excite and receive Lamb signals without mutual influence. The miniaturized LCT produced is shown in Figure 22b. The length × width of the probe was 16.0 mm × 12.0 mm.

**Figure 23:**
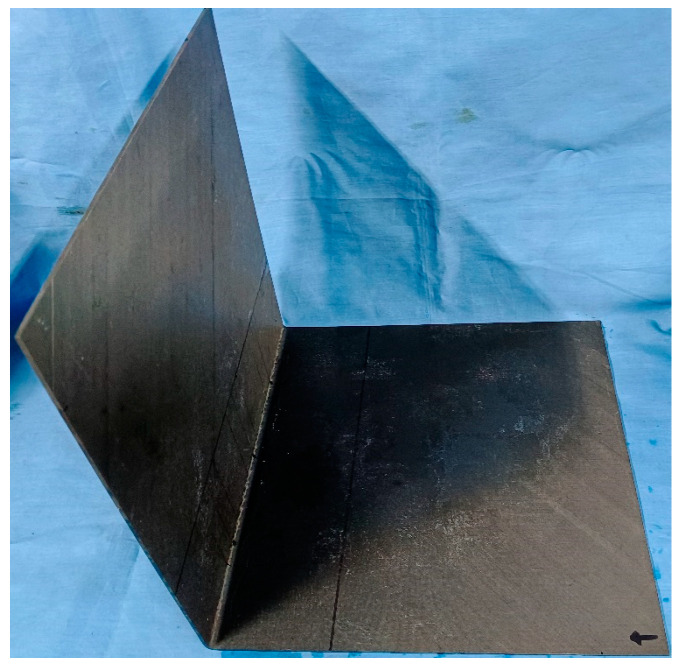
CFRP bending plate specimens (the arrow represents the positive direction of the *X*-axis).

The prepared CFRP bending specimen is shown in Figure 23. The bending angle of the specimen was 90°, the bending radius was 5.0 mm, and the length × width of the plate area of the CFRP bending plate was 300.0 mm × 300.0 mm, with a thickness of 2.0 mm. Delamination damage with dimensions of Φ6.0 mm, Φ10.0 mm, and Φ15.0 mm were prefabricated in the CFRP bending plate specimens. Delamination damage was established by filling anti-sticking paper in the middle layer of the CFRP bending plate specimens.

**Figure 24:**
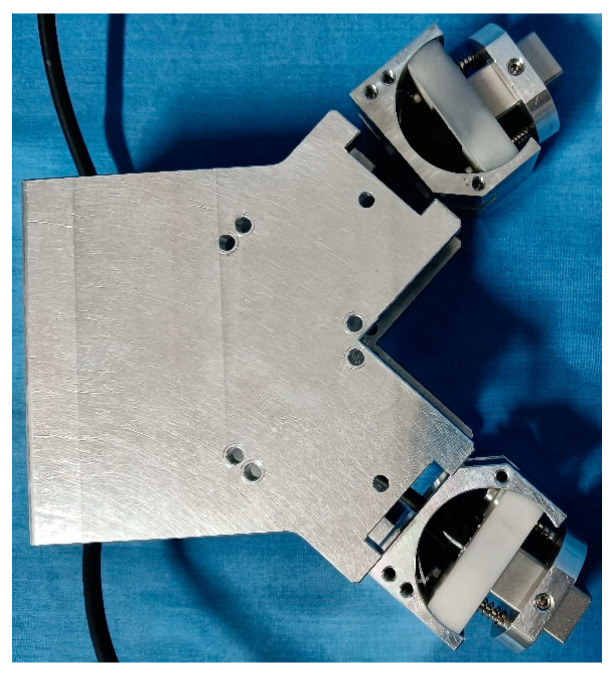
Probe clamping device.

In the experiment, changes in the position of the sensors (including the distance and relative orientation between the two sensors) would have seriously affected the detection results of delamination damage. As shown in Figure 24, a comb-shaped transducer probe clamping device was designed based on the structural characteristics of CFRP bending plate specimens. The clamping device ensured that the distance and relative orientation between the probes were consistent during each detection. The clamping device’s built-in spring and retractable configuration guaranteed a consistent coupling force between the probe and the CFRP bending plate throughout each detection operation.

**Figure 25:**
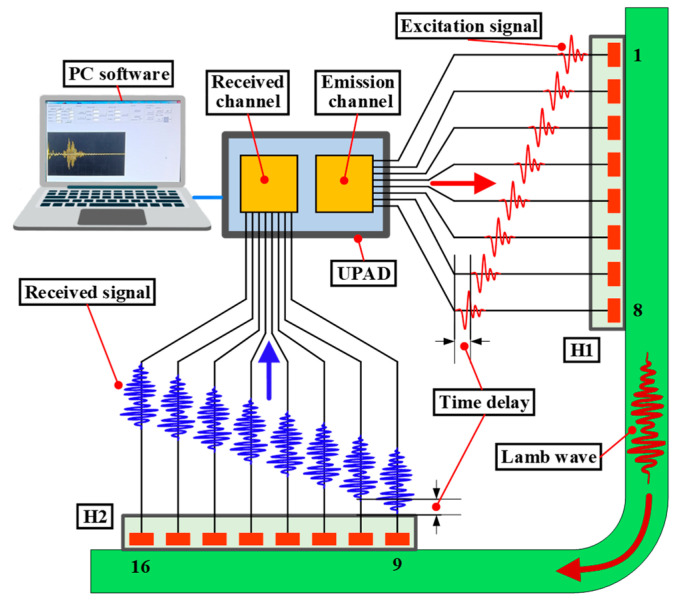
The Lamb wave phased array detection system (the arrow represents the direction of the Lamb wave signal).

The Lamb wave phased array detection system is shown in Figure 25. The Lamb wave phased array detection system consisted of an ultrasonic phased array detector (UPAD) with 32 channels, a PC, a miniaturized LCT (including a clamping device), and a CFRP bending plate specimen. The PC interacted with UPAD through the USB interface, which could control the time delay of each array element and transmit Lamb wave signal data. The verification experiment was carried out using the pitch–catch method. The 1^st^ to 8^th^ channels of UPAD were controlled by the PC to generate the excitation signal with a set time delay, which was employed to excite the 1^st^ to 8^th^ array elements of probe H1. The Lamb waves excited by probe H1 were propagated in the CFRP bending plate and were then perceived by probe H2. The Lamb wave signals were received by channels 9 to 16 of UPAD with a set time delay. The A0-mode Lamb wave signal after phase-controlled mode focusing was obtained.

**Figure 26:**
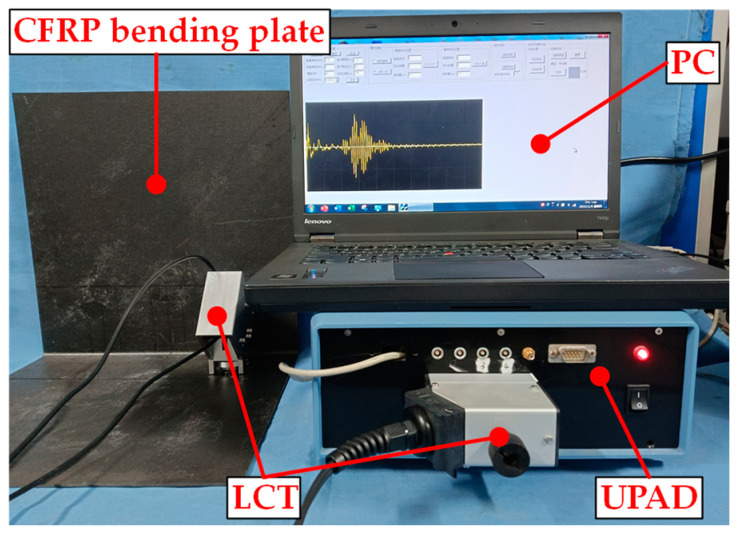
Experimental system.

The experimental system is shown in Figure 26. The A0-mode Lamb wave was excited to detect delamination damage in the CFRP bending plate. The sampling rate of UPAD was 30.0 MHz, and the sampling duration of the Lamb wave signal was 150 μs. Throughout the experiment, the data were gathered on ten occasions and subsequently averaged to mitigate the impact of noise interference. The detection results would have been affected by the potential variations in environmental and operational conditions, along with sensor degradation. It was necessary to consider some issues that may have affected non-destructive testing to avoid the impact of these factors on the detection results. The research experiment was conducted in an environment where the temperature fluctuated by less than 5 °C. The configuration of each detection experiment was consistent. The total time consumed for all detection experiments was within 5 h, thereby mitigating the influence of sensor degradation on the detection. An experiment on array elements without time delay was carried out to verify the effectiveness of the miniaturized LCT in controlling Lamb wave modes, and the time delay of all excitation and receiving array elements of the miniaturized LCT was set to 0.0.

**Figure 27:**
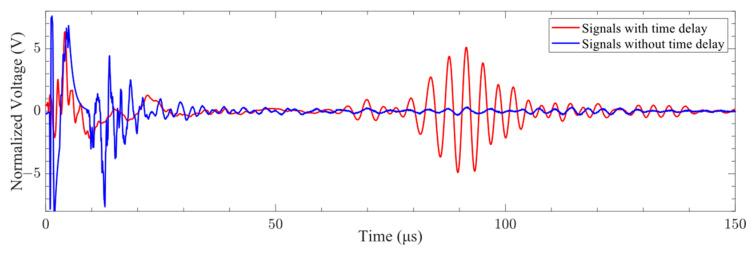
Signals with and without time delay.

The signals generated by the miniaturized LCT with and without time delay are shown in Figure 27. It can be seen from the figure that the signals generated by the miniaturized LCT with time delay were superimposed into the A0-mode Lamb wave in the time range of 70 to 120 μs. The signals generated by the miniaturized LCT without time delay were not superimposed into a fixed-mode Lamb wave throughout the time domain. It has been proven that a miniaturized LCT can effectively control the mode of Lamb waves by adding a time delay.

**Figure 28:**
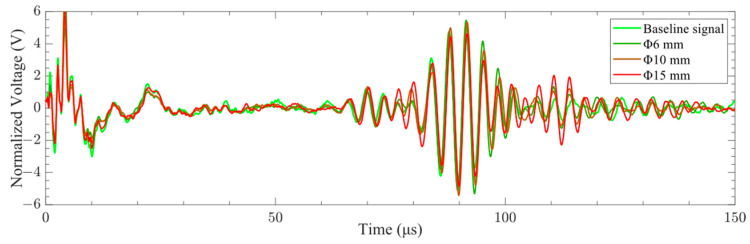
Time domain signals of A0-mode Lamb waves with different delamination damage sizes.

The A0-mode Lamb wave signals of different delamination damage sizes were obtained in the experiment, as shown in Figure 28. It can be seen from the figure that the characteristics of the Lamb wave signal were insignificantly affected by the delamination damage size. The phase and amplitude of the Lamb wave signal were essentially constant under different delamination damage sizes. Therefore, the *SDC* was used to evaluate the difference in the signals.

**Figure 29:**
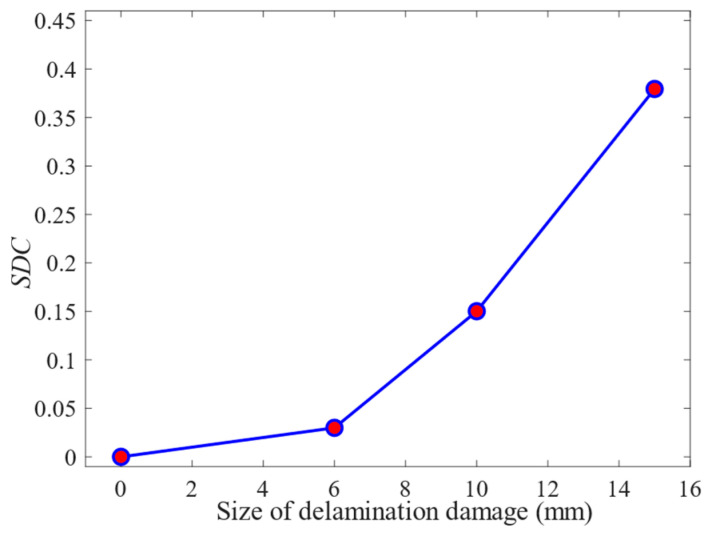
The *SDC* values of different delamination damage sizes.

The Lamb wave signals from 60 μs to 140 μs were processed to obtain the *SDC* values of different delamination damage sizes, as shown in Figure 29. It can be seen from the figure that the *SDC* value increases with the increase in the delamination damage size. The results of the comparison experiments and simulations show that the finite-element method can accurately simulate the propagation of Lamb waves in CFRP bending plates. Delamination damage can be quantified according to the variation rule of *SDC* values with the size of the delamination damage.

## 5. Quantification of Delamination Damage

**Figure 30:**
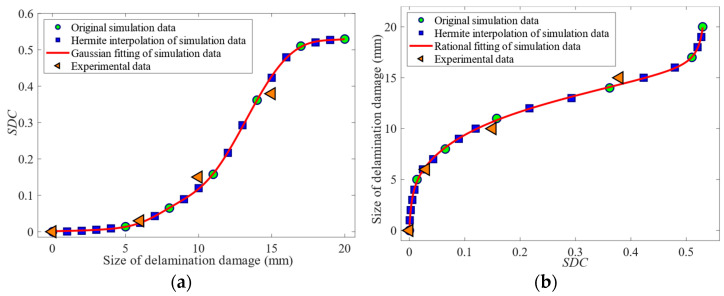
The relationship between delamination damage size and SDC values: (**a**) Gaussian fitting; (**b**) Rational fitting.

Only seven kinds of *SDC* values of delamination damage sizes were provided by finite-element simulation. To better understand the law of *SDC* values with the delamination damage sizes, more sample data were required for analysis. Piecewise cubic Hermite interpolation was applied to simulation data to obtain more *SDC* values of delamination damage. The Hermite interpolation results of the simulation data are shown in Figure 30. Based on the Hermite interpolation results of simulation data, Gaussian and Rational functions were employed to fit the relationship between the delamination damage sizes and *SDC* value. The Gaussian and Rational fitting expression is shown in Equations (6) and (7), where *x* represents the delamination damage size, and *y* represents the *SDC* value of the delamination damage signal. The Gaussian and Rational fitting accuracy parameter *R*^2^ stood at 0.99992 and 0.99962, with the corresponding fitting curve depicted in Figure 30. There was a high degree of fitting between the size of the delamination damage and the *SDC* value. The *SDC* value of the experimental data is shown in Figure 30. It can be seen from the figure that the changing trend of the *SDC* value of the experimental data with the delamination damage size is the same as the simulation results. When the delamination damage size was less than Φ6 mm, the *SDC* value increased slowly with the increase in the delamination damage size. The *SDC* value was roughly linear with the delamination damage size in the range of Φ6~Φ15 mm, and the *SDC* value increased significantly with the increase in the delamination damage size. The variation laws observed in the experimental and simulation data hold significant value for delamination damage quantification.

(6) $$y=0.636e^{\frac{{\left(x-27.42\right)}^{2}}{11.06}}+0.023e^{\frac{{\left(x-8.44\right)}^{2}}{2.192}}+0.258e^{\frac{{\left(x-15.98\right)}^{2}}{4.692}}$$

(7) $$x=\frac{-37.44y^{3}+18.46y^{2}+1.013y+0.0001214}{y^{4}-2.989y^{3}+0.9713y^{2}+0.1882y+0.0008674}$$

To verify the accuracy of the fitting expression for the quantification of delamination damage, the difference between the calculation results of the fitting expression and the experimental data was analyzed. According to Equation (6), the sequence *S* of the delamination damage sizes and their corresponding *SDC* values were obtained. Then, based on the *SDC* values of the experimental data, we queried the delamination damage sizes corresponding to similar *SDC* values in sequence *S*. In addition, according to Equation (7), the delamination damage size could be directly calculated based on the *SDC* value of the experimental data. The absolute and percentage errors of the delamination damage quantification using the fitting expression are shown in Table 6. It can be seen from the table that the absolute error of the delamination damage quantification with Gaussian and Rational fitting expression did not exceed 0.8 mm and 0.7 mm, and the percentage error was not more than 8% and 7%. The Gaussian and Rational fitting methods exhibited a considerable degree of consistency in accurately determining the delamination damage size. However, compared with the Gaussian fitting method, the Rational fitting method is more convenient to calculate the delamination damage size. This study indicates that the A0-mode Lamb wave can accurately quantify delamination damage.

## 6. Conclusions

In this research, the Lamb wave detection of delamination damage in CFRP bending plates using finite-element and experimental methods was studied. The main work and conclusions are as follows:

(1) The effect of the linear array PZT phase time-delay method on Lamb wave mode control was investigated by finite-element simulation. The phase time-delay method realizes the control and enhancement of a single-mode Lamb wave, which can excite A0-mode and S0-mode Lamb waves, respectively. The effectiveness of the phase time-delay method for Lamb wave mode control was proved.
(2) The finite-element models of delamination damage with different sizes were established, and the delamination damage was detected using A0-mode and S0-mode Lamb waves. The detection results indicate that compared with the S0-mode Lamb wave, the A0-mode Lamb wave exhibits high sensitivity to delamination damage. The strong correlation between *SDC* and the size of delamination damage can be utilized to quantify delamination damage.
(3) Based on the conclusion of finite-element simulation, a one-dimensional miniaturized LCT was developed to excite the A0-mode Lamb wave for the detection of delamination damage in the CFRP bending plate. The experimental results verify the correctness of the simulation results.
(4) Based on the Hermite interpolation results of simulation data, Gaussian and Rational functions were employed to fit the relationship expression between delamination damage size and *SDC*, which can accurately quantify delamination damage. The absolute error of the delamination damage quantification with Gaussian and Rational fitting expression does not exceed 0.8 mm and 0.7 mm, and the percentage error is not more than 8% and 7%.
(5) The detection and signal processing methods employed in the present research are easy to operate and implement, and accurate delamination damage quantification results have been obtained. The experimental and modeling methods, signal analysis techniques, and fitting expressions presented in this article can be used as a reference for detecting delamination damage in CFRP.
(6) In the future, further research will be conducted on the influence of the relative position between sensors and delamination damage on the quantification of delamination damage.

## Figures and Tables

**Table 1:** The properties of CFRP materials in the numerical simulation study.

*E*_1_ (GPa)	*E*_2_ = *E*_3_ (GPa)	*G*_12_ = *G*_13_ (GPa)	G_23_ (GPa)	*ν*_12_ = *ν*_13_	*ν*_23_	*ρ* (kg/m^3^)
280.0	10.0	4.0	2.0	0.28	0.45	1639.0

**Table 2:** The stiffness coefficient of PZT-5H.

*D*_1111_ = *D*_2222_ (MPa)	*D*_3333_ (MPa)	*D*_1122_ (MPa)	*D*_1133_ = *D*_2233_ (MPa)	*D*_1212_ = *D*_1313_ (MPa)	*D*_2323_ (MPa)
127,205.0	117,436.0	80,212.2	84,670.2	22,988.5	23,474.2

**Table 3:** The piezoelectric coefficient of PZT-5H.

*d*_3 11_ = *d*_3 22_ (10^−12^ C/N)	*d*_3 33_ (10^−12^ C/N)	*d*_2 12_ = *d*_1 13_ (10^−12^ C/N)
−274.0	593.0	741.0

**Table 4:** The relative dielectric constant of PZT-5H.

*D*_11_	*D*_22_	*D*_33_
1704.4	1704.4	1433.6

**Table 5:** The simulation results and theoretical calculation results of group velocity.

	A0	S0
simulated group velocity (m/s)	1235.7	4369.2
theoretical group velocity (m/s)	1302.1	4359.4
absolute error (m/s)	66.4	9.8
percentage error (%)	5.1	0.2

**Table 6:** The error of the fitting expression to the delamination damage quantification.

Delamination Damage Size (mm)	Measured Value (mm)		Absolute Error (mm)		Percentage Error (%)	
	Gaussian	Rational	Gaussian	Rational	Gaussian	Rational
6.0	6.3	6.3	0.3	0.3	5.0	5.0
10.0	10.8	10.7	0.8	0.7	8.0	7.0
15.0	14.3	14.3	0.7	0.7	4.7	4.7

## Data Availability

Data are contained within the article.
